# Contraceptive method use trajectories among young women in Kenya: A qualitative study

**DOI:** 10.3389/fgwh.2022.973971

**Published:** 2022-09-13

**Authors:** Lisa M. Calhoun, Mahua Mandal, Bernard Onyango, Erick Waga, Courtney McGuire, Eliya M. Zulu, Thomas van den Akker, Lenka Benova, Thérèse Delvaux, Ilene S. Speizer

**Affiliations:** ^1^Carolina Population Center, University of North Carolina at Chapel Hill, Chapel Hill, NC, United States; ^2^Athena Institute, Vrije Universiteit, Amsterdam, Netherlands; ^3^Department of Public Health, Institute of Tropical Medicine, Antwerp, Belgium; ^4^African Institute for Development Policy, Nairobi, Kenya; ^5^Department of Obstetrics and Gynecology, Leiden University Medical Center, Leiden, Netherlands; ^6^Department of Maternal and Child Health, Gillings School of Global Public Health, University of North Carolina at Chapel Hill, Chapel Hill, NC, United States

**Keywords:** family planning, method choice, youth, contraception, life course

## Abstract

**Background:**

Many young women experience important key life transitions during adolescence and early adulthood, such as initiation of sexual activity, first use of contraceptives, marriage, and childbirth. For young women to be able to plan and manage their lives, it is critical to understand how these life events intersect and shape their contraceptive decision-making. This study aims to explore young women's contraceptive method use trajectories, including the factors that influence contraceptive decision-making throughout adolescence and youth.

**Methodology:**

In 2019, the Full Access, Full Choice project (FAFC), implemented by the University of North Carolina at Chapel Hill and the African Institute for Development Policy, conducted 30 in-depth interviews with young women aged 18–24 years in three counties in Kenya (Nairobi, Mombasa and Migori). Eligible respondents had used two or more modern contraceptive methods. Interview guides utilized a modified life history approach to capture details about respondents' contraceptive use and life experiences from the time they first used contraception until the time of interview.

**Results:**

We identified five separate contraceptive use trajectories based on the occurrence and timing of marriage, childbirth, and contraceptive method choice as well as various influences on contraceptive decision-making. The majority of respondents began their contraceptive journey by using male condoms or emergency contraception, but subsequent contraceptive decisions were varied across trajectories and influenced by different factors. For many women, the initiation of a non-coitally dependent method occurred after the birth of a child; for some, this was the first method used. Once women transitioned to using a non-coitally dependent method such as injectables or implants, many cycled through different methods to find one that had fewer side effects or provided the desired duration of protection.

**Discussion:**

This study highlights the nuanced needs of young women throughout their adolescent and youth years in Kenya. This suggests that programs and policies need to encompass young women's diversity of experiences and motivations to best serve them.

## Introduction

Family planning (FP) has numerous health and social benefits for women and families (1–4). These are particularly salient for young women who are at the beginning of their reproductive life course and have the desire and need to delay or space pregnancies (5, 6). Important reproductive health transitions such as initiation of sexual activity, contraceptive use, the experience of a first pregnancy or birth, and union formation often take place during the adolescent and youth years (between ages 10–24 years) (1, 2, 7–9). The reproductive health transitions are often accompanied by changes in school enrollment, employment and other forms of economic activity, and housing status (1, 2). It is critical to understand how these numerous life events intersect and shape contraceptive decision-making for young women in order to strengthen FP programming that better meets the varying needs of young people.

The factors that influence contraceptive use (including uptake, switching, discontinuation and contraceptive method choice) among young women are multifactorial. Numerous barriers and facilitators to contraceptive decision-making have been described in many countries across sub-Saharan Africa and include factors related to knowledge about contraception (10–12), social environment (13–16), partner dynamics (10, 11), concerns about side effects and method characteristics (11, 12, 17), as well as factors related to the supply environment, such as accessibility, privacy, cost, and method availability (10, 16, 18–20). These factors influence whether young women or their partners use contraception or not, the circumstances under which they use contraception, what method young women and their partners use, when they adopt a method, or how long they use the method before discontinuing or switching.

To the best of our knowledge, research is limited on why and how contraceptive method choices change across the adolescent and youth years. Yet, it is widely accepted that contraceptive use is dynamic and young women may initiate, change methods and discontinue over short periods of time (21–24). This, coupled with the multitude of life changes young women experience, points to the need to utilize holistic, life course approaches similar to those that have been used in work among women of all ages (25–27) to examine young women's decision-making around contraceptive adoption and method choice. This knowledge can inform the development of program strategies seeking to provide targeted information and services on reproductive health and contraception to young women. In particular, understanding changes in circumstances over the adolescent and youth years can inform how, where, and when to intervene programmatically to meet the changing needs and desires of young women.

This study explores young women's contraceptive use trajectories, including the factors that influence contraceptive decision-making throughout adolescence and youth among a sample of young women who have used two or more modern contraceptive methods. Using qualitative data from young women in Kenya that asks them about their contraceptive decision-making from the time they first used contraception until the time of the study, we take a holistic approach to understanding young women's contraceptive journeys and how key life events, such as marriage and childbirth, influence use and choice. Building on these key life events, we identify different typologies of young women, which can be useful in the development of program strategies that enable them to access and use contraceptive methods of their choice at various stages of their lives.

## Materials and methods

### Full access, full choice project

This study was undertaken as part of the Bill & Melinda Gates Foundation (BMGF) funded Full Access, Full Choice project (FAFC), which aims to generate and synthesize evidence on expanded contraceptive method choice for adolescents and youth globally, but with a focus on Kenya and Niger. FAFC is a collaboration between the Carolina Population Center at the University of North Carolina at Chapel Hill (UNC) and two non-profit research institutes: the African Institute for Development Policy (AFIDEP) in Kenya and GRADE Africa in Niger.

In 2018, technical workshops were held in Washington, D.C., Niamey, Niger and Nairobi, Kenya which sought to identify key evidence gaps in expanded contraceptive method choice for adolescents and youth, both globally and at the country levels. The main output for each meeting was a learning agenda containing key research priorities and learning questions (https://dataverse.unc.edu/dataverse/fafc).

In Kenya, the site of this study, the project works in focal counties (Nairobi, Mombasa, Migori, West Pokot and Wajir) chosen based on availability of secondary data sources, levels of contraceptive use among young people, presence of adolescent and youth-focused implementing partners, advocacy partners, regional representation, and political commitment to ensuring access to FP for young people. The counties were selected in collaboration with the Kenya Ministry of Health (MOH) and the Kenya National Council for Population and Development (NCPD).

### Context

Kenya is one of the fastest growing economies in sub-Saharan Africa, with an average economic growth of 5.7% from 2015 to 2019 (28). In 2019, the Kenyan population was estimated to be 47.6 million, with children and young people under the age of 25 comprising 60% of the total population (29).

Research from Kenya shows that women's transition to first sexual activity typically occurs at an average age of 18.4 years in urban areas and 16.7 in rural areas, the average age at marriage is 22.5 years in urban areas and 20.8 years in rural areas, and the average age at first pregnancy is 20.3 years with variability by residence and region (9). The COVID-19 pandemic intensified concerns about the adolescent pregnancy rate in Kenya, and evidence shows higher rates of adolescent pregnancy amongst adolescents whose schools were closed as part of the COVID-19 lockdown compared to a pre-pandemic cohort of their peers (30). The nuanced needs of young women in Kenya are reflected by estimates of contraceptive use and method choice that tend to differ by several demographic factors, including marital status, religion, residence, and economic status (9, 31, 32). For example, modern contraceptive use among married women aged 15–24 was 56% in 2018; compared to 48% among unmarried sexually active young women (9). Further, there are differences in method choice with married young women and those with children being more likely to use longer acting methods (e.g., implants) whereas adolescents and young women without children tend to use short-acting methods [emergency contraception (EC), condoms, and injectables] (9, 20, 31, 33).

Kenya has a hierarchal public health service structure with hospitals, health centers, dispensaries and community health volunteers, as well as a robust private sector with hospitals, clinics, pharmacies and informal drug outlets. Contraception should be available free of charge at public sector facilities in Kenya, though Radovich et al. found that up to half of modern users made out of pocket payments when obtaining their method from a public facility (34). About 63% of married women ages 15–24 sourced their contraceptive method from the public sector compared to 35% of unmarried women (9). This partly reflects the differences in method choice among these two groups, as longer acting methods are available for free or low cost at public facilities, whereas many of the unmarried young women are likely sourcing short-acting methods from pharmacies (20).

In 2015, the Government of Kenya released the National Adolescent Sexual and Reproductive Health Policy, which is aimed at creating a framework to ensure young Kenyans have access to the appropriate knowledge and care they need to achieve their reproductive goals (35). The policy includes eight cross-cutting objectives focused on addressing specific harmful practices and behaviors, promotion of rights for young people, meeting needs of marginalized and vulnerable adolescents, and increasing access to information and age-appropriate comprehensive sexuality education (CSE). A guiding principle of the policy is to be responsive to the varying sexual and reproductive health needs of adolescents.

### Study design

This study uses data from in-depth interviews (IDI) to understand adolescent girls' and young women's contraceptive trajectories. This research topic was selected as a high priority learning question at both the global and Kenya technical workshops. The technical workshops were convened to understand critical policy-relevant evidence gaps to ensure that the project's research was relevant to local realities and needs. To strengthen the design of this study, we created technical advisory groups in Kenya and the United States of America that supported refinement of the research questions and reviewed finalized study materials. The global technical advisory group comprised of individuals from international non-governmental organizations (NGOs), BMGF, UNC, and AFIDEP. The Kenya technical advisory group comprised of individuals from NGOs, University of Nairobi, Ministry of Health (MOH; both central and county-level officers), NCPD, and AFIDEP. The Kenya participant list included youth representatives from civil society groups in order to ensure that the youth perspective was considered. From the list of five priority FAFC counties, Nairobi, Mombasa and Migori counties were selected as study sites because contraceptive use among young people was higher in these counties than Wajir and West Pokot, which was important for meeting our eligibility criteria.

#### Sampling and recruitment

Study participants were recruited from service delivery points (SDP)—both health facilities and pharmacies—in the three target counties. To facilitate selection of health facilities, Kenya Health Information System data were reviewed to identify health facilities with high family planning client volumes, including adolescent and youth clients. The MOH sub-county reproductive health coordinators reviewed the list of health facilities with large client loads and gave recommendations on which facilities would be suitable recruitment sites based on their local knowledge of client populations. Two to three public facilities, two private facilities and one pharmacy were selected in each county for a total of sixteen SDPs. Among the public facilities, two public health centers were included in Nairobi, a public dispensary and a health center in Mombasa, and three public hospitals were included in Migori.

The recruitment of study participants was undertaken by county (Nairobi, Mombasa and Migori) and parity (no children and 1+ child). The aim was to undertake ten IDIs per county: five with nulliparous women and five with women who had one or more children. The number of respondents by county and parity are shown in Table 1.

**Table 1:** Participant characteristics at time of interview.

**Characteristic**	**Number of women**
**County of residence**	
Nairobi	10
Mombasa	9
Migori	11
**Recruitment site**	
Public hospital	7
Public health center	8
Public dispensary	2
Private health facility	8
Pharmacy	5
**Age**	
18–21 years	13
22–24 years	17
**Highest level of education completed**	
Partial or completed primary	9
Partial or completed secondary	10
Partial or completed post-secondary	11
**Employment/Income generation**	
None	12
Employed or generates income	13
Current student	5
**Marital status**	
Married	18
Not married	12
**Parity**	
Nulliparous	15
1+ child	15
Number of contraceptive methods ever used^*^	
2	15
3	11
4	4
**Type of method ever used (multiple responses possible; all modern methods reported are listed)**	
Male condoms	21
EC	15
Oral pills	8
Injectable	21
Implant	15
Total number of respondents	30

* Only users of two or more methods were included in this study.

Young women were eligible for this study if they were between 18 and 24 years of age and had ever used two or more contraceptive methods that could be obtained from an SDP: implant, intrauterine device (IUD), injectable, oral pills, EC, male condoms, or female condoms. Use of traditional methods was not a focus of this study. We chose to focus on women 18 and older due to ethical issues related to studying minors. Additionally, since we targeted women with a varied contraceptive history, it would have been relatively difficult to find and recruit girls <18 years who had used two or more contraceptive methods. Respondents did not need to be using a contraceptive method or be seeking FP services at the time of recruitment; respondents could have been at the SDP for another service.

#### Study procedures

Prior to the start of data collection, a 10-day data collector training was held in Nairobi, Kenya, explaining study design, types of contraceptive methods, ethics, and interviewing techniques. Data collectors also conducted mock interviews during the training and reviewed and refined the translation of the interview guides. Sessions were facilitated by investigators from UNC and AFIDEP. All interviewers were fluent in the respective local languages for the sites where they would be collecting data, were under the age of 30, and were experienced interviewers who had worked on previous qualitative studies on adolescent and youth sexual and reproductive health. Interviewers were trained to build rapport with respondents through use of common grounding behavior prior to the interview, identification of a site for interview where the respondent was comfortable, and being courteous and attentive during the interview.

The data collection activities were led by AFIDEP. After conducting a pilot of in-depth interviews in two Nairobi SDPs, study data were collected between August and September 2019. Permission from health facility managers and pharmacy owners was obtained before the start of data collection. Participants were recruited from waiting areas of sampled facilities and outside of pharmacies. A checklist was employed by interviewers to determine if potential participants met the eligibility criteria, and for eligible participants, a verbal informed consent process was undertaken. The interviews were conducted in a private setting in the preferred language of the respondent (English, Kiswahili, DhoLuo, or Kuria) immediately after informed consent was obtained. All interviews were digitally recorded and transcribed verbatim.

#### Data collection instrument

We used a modified life history approach to understand adolescent girls' and young women's family planning history and decision-making processes. The life history method is a qualitative approach to identifying and documenting information about an individual's decision or influences over time and how these relate to current attitudes or behaviors (36). The research team jointly developed a semi-structured IDI guide in English that asked about the participants' life circumstances, including their education, living arrangement, employment, relationships, children, and pregnancies. Data were also collected to understand participant's trajectories through their contraceptive journeys, including: the types of contraceptive method they used, starting with the first method ever used; reasons for choosing the method, including life circumstances, characteristics of the method and the types of people that influenced their decision; the type(s) of partners they were with while using the method; other methods they may have considered using at the time; characteristics of the method they liked and disliked; and use of dual methods. The IDI guide was pre-tested and revised before translating it into Kiswahili, DhoLuo, and Kuria. A meeting was held with a group of Kenyan female youth to review the guides prior to finalization. Final revisions to the guide were made based on the interviewers' observations and experiences during piloting.

#### Data analysis

Following transcription and translation of IDIs into English, data were uploaded and coded in Dedoose software (v.8.3). Using the *coding reliability* approach of thematic analysis, a priori codes were created based on the IDI guide (37). Five members of the research team at AFIDEP and UNC read the same two transcripts and through group discussions agreed upon additional codes based on emergent themes. To establish coding standards, team members double-coded sections of a third transcript and assessed intercoder reliability. Discrepancies in coding were resolved through discussion and the coding framework was adjusted accordingly. The remaining transcripts were divided among team members. The lead coders periodically checked the validity of code applications on randomly selected transcripts.

Matrices were developed to capture the timing of key life events, such as relationship status, pregnancy and childbirth, with respect to each contraceptive method used and the influences on method choice. In the matrix, women were assigned the main method they were using and dual use was indicated, where applicable. Transitions in method use occurred when a woman stopped using her main method and subsequently started using another method or discontinued all together. Common contraceptive trajectories were identified by reviewing and interpreting the occurrence and timing of life events, choice of contraceptive methods, and the factors within their life course that influenced their choices.

#### Ethics approval

Approval for the study protocol, informed consent procedures, and materials and survey tools were provided by the AMREF Health Africa Ethics and Scientific Review Committee (P205/2019), National Commission for Science, Technology and Innovation in Kenya, and the University of North Carolina at Chapel Hill Institutional Review Board (19-1360). Additional approvals were secured from each county's Director of Health.

## Results

### Characteristics of respondents

In total, 30 young women recruited across the 16 SDPs met study inclusion criteria and participated in the in-depth interviews. Table 1 presents the characteristics of the respondents at the time of interview. Thirteen participants were in the age group 18–21 and 17 were ages 22–24. About one-third of the women were from each of the three counties, Nairobi, Mombasa and Migori. Seventeen were recruited from a public health facility, eight from private health facilities, and five from pharmacies. The respondents' levels of education ranged from partial primary school to having completed post-secondary school. Thirteen women were employed or engaged in an income-generating activity and five were students at the time of interview. Eighteen women were married. Half of the women were nulliparous. Additionally, 15 had ever used two methods, 11 had ever used three methods and 4 had ever used four methods. Condoms (*n* = 21) and injectables (*n* = 21) were the most frequently reported methods ever used (Table 1).

### Contraceptive use trajectories

**Figure 1:**
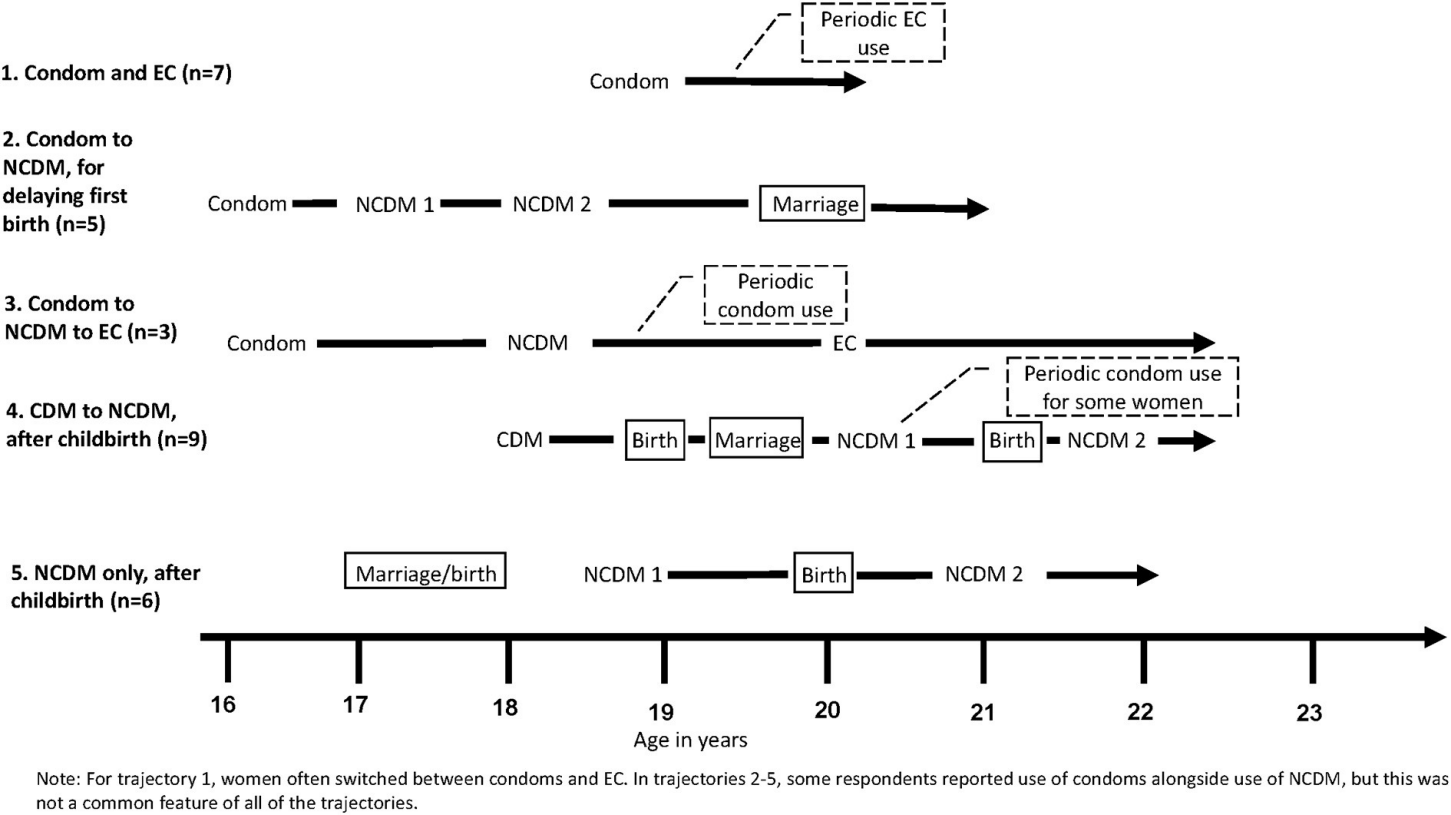
Five trajectories to contraceptive use and method choice.

Five main trajectories were identified from the in-depth interviews based on patterns in respondents' characteristics, decision-making influences, and method choice as well as the timing of these (Figure 1). Trajectory one is characterized by young women who first use a condom and continue condom use with periodic EC use. Trajectory two includes respondents who start with condoms and then transition to non-coitally dependent methods (NCDM) such as injectables, implants and oral contraceptive pills in order to delay their first birth. Trajectory three includes women who start with condoms and then transition to NCDM, followed by EC. Trajectory four is characterized by respondents who start with either condoms or EC, and then transition to a NCDM for the purpose of child spacing. Trajectory five includes respondents who only used NCDM after childbirth. Each respondent was placed in the trajectory that most closely resembled her sequence and timing of life events, including the contraceptive methods used and the factors related to changes in method choice over time.

Additionally, two categories of method types emerged from the data. Use of male condoms or EC are referred to as coitally-dependent methods (CDM) and use of implants, injectables and oral pills are referred to as NCDM. When a CDM can be specified because all respondents used that specific method at that time, such as condoms, then we write the name of the method rather than CDM. In Figure 1, each line represents a single trajectory with the starting point being the average age at first contraceptive use and the end point being the average age at interview for that specific trajectory (indicated by the arrow head). Therefore, the length of each line represents the average duration of respondents' contraceptive history. The sequencing of key events is indicated on the trajectory with words describing the events; key events include initiation of a method, a birth, or marriage. Periodic use of specific methods is called out in a dotted box. Each line does not reflect method duration of use or continuation. Of note, the lines represent the general pattern of the events, but the specific details may be different for some study participants categorized into the trajectory. Each of the trajectories are described in more detail below and include the defining characteristics and thematic influencing areas on the women, and factors related to transitions in contraceptive use over time.

Table 2 describes the characteristics of the respondents in each of the five contraceptive use trajectories. Respondents in trajectories 2 and 3 intitiated first contraceptive use at a younger age on average than women in trajectories 1, 4, and 5. Women in trajectories 1 and 2 were nulliparous when they began using contraception and remained so at the time of interview. In constrast, all of the women in trajectory 5 had at least one child when they began using contraception, and all had at least a second child by the time of interview. In trajectories 1 and 3, respondents were unmarried at first contraceptive use and remained so at the time of interview. The majority of respondents in trajectories 2 and 4 transitioned from being unmarried at first contraceptive use to being married at the time of interview. The average number of modern FP methods used ranged from 2.0 among respondents in trajectory 1 to 3.3 in trajectory 3.

**Table 2:** Characteristics of respondents in each trajectory.

**Trajectory**	**Number of women**	**Average age (years)**		**Average parity**		**Number of women who are married**		**Average number of modern FP method used**
		**At first use**	**At time of interview**	**At first use**	**At time of interview**	**At first use**	**At time of interview**	
1: Condoms and EC	7	19.0	20.7	0.0	0.0	0	0	2.0
2: Condom to NCDM	5	16.2	21.0	0.0	0.0	0	3	2.6
3: Condom to NCDM to EC	3	16.3	22.3	0.0	0.33	0	0	3.3
4: CDM to NCDM	9	18.3	22.4	0.1	1.6	1	9	2.8
5: NCDM only	6	18.5	22.0	1.0	2.0	4	6	2.3

### Condom or EC: A common starting point for contraceptive use

A defining feature of the trajectories was which method the respondent started with and what influenced her to start with that method. After the first method used, the trajectories differentiated, and the role of the influences and thematic constructs becomes more divergent, nuanced, and multifactorial.

Across all respondents, the majority began their contraceptive journey with condoms (*n* = 21) or EC (*n* = 2) with only six respondents starting with a non-coitally dependent method (all in trajectory 5 where they started after having a child). Due to the similarity in first method use of condoms and EC, we will discuss the influences on the first use of condoms or EC before further elaborating the differences across trajectories.

Young women who began their contraceptive journey with condoms or EC most frequently cited reasons related to the thematic areas of knowledge, accessibility, couple dynamics, low cost and fertility desires. Most women shared that they first used condoms with their boyfriends early in the relationship, primarily because condoms provide dual protection from pregnancy and sexually transmitted infections (STI). When sharing that she chose to use a condom as her first method with her boyfriend, one woman stated the following:

*Respondent (R): “…I did not want to get pregnant or those diseases like STIs and HIV/AIDS.”*

*Interviewer (I): “Who advised you? Were you advised by anybody or you just decided by yourself?”*

*R: “We were taught in school to use protection during sex.”*

21-year-old, Migori, nulliparous

A few women explicitly spoke to the fact that initially they did not trust their partners and were cautious about both becoming pregnant with such partners, as well as contracting STIs, including HIV from them:

*I: Why did you decide to use a condom with this partner?*

*R: Okay at first, you know, at first you are never sure of someone you love so…I used it [a condom] because I had some trust issues at first…I was not sure of him…Maybe the previous girl... you know…[had] STIs..*.

*I: Okay, okay you were protecting yourself from STIs*.

*R: You know when it is your first time to have sex, it is not even STIs or HIV, it is pregnancy and the fact that I am still living with my mum of course she threatens me: 'I don't want to see you being pregnant here, yeah... definitely I used it... sincerely speaking I used it to prevent getting pregnant*.

19-year-old, Nairobi, nulliparous

Women commonly stated they had no knowledge of other methods when they decided to use a condom and first learned about condoms from their boyfriends, friends, and school-based life skills lessons. Some women reported that their family members influenced their decision to use a condom. One woman shared that multiple family members had encouraged her to use a condom at first sex:

“*My dad. My dad used to tell me that if I were to sleep with a man, I should always use protection before we get to know each other. So, I just had it at the back of my mind. Any time anyone approached me, I would think about it. My grandmother also told me the same thing. ‘The day you meet a man, don't have sex without protection. Make sure you use it.' So, I heeded their advice.”*

24-year-old, Migori, 2 children

Additionally, many women said that they chose to use condoms because they were easily accessible and inexpensive at pharmacies and shops. One woman explained how having no knowledge about other methods in addition to condoms being easy to obtain made it the “default” method choice for her first method:

“*It [the condom] is not because I wanted the method, but because it was the easy one to get at that time and I did not have knowledge of those others.”*

21-year-old, Migori, nulliparous

Most respondents indicated that the decision to use condoms was mostly theirs, and that their boyfriends agreed with the decision even if they did not especially enjoy using condoms. Women often shared that their boyfriends were “okay with condoms” and that the boyfriends “understood” when the women raised and discussed condom use. One woman stated her boyfriend “did not complain…though mostly he does not want to use condoms.” She went on to explain, however, that her boyfriend was the one who had procured condoms the first time they used it.

Several women also expressed that condoms were better for young people to use and that hormonal methods of FP were for married women or were for after giving birth. This belief was rooted in concerns that use of hormonal methods may impact their fertility. One woman shared about her choice to use condoms:

*I: Had you heard of other methods [other than condom]?*

*R: I had heard about other methods but I didn't want to use them before I get a child*.

*I: Why?*

*R: Because you may decide use a family planning and fail to give birth again once you've got someone to marry you; then you start blaming God for having not given you the kids yet you are the one who have killed them yourself*.

22-year-old, Mombasa, 1 child

The two women who started their contraceptive journey with EC first learned about EC from their boyfriends. One 21 year old woman from Migori with one child who used EC as her first method shared that she was afraid of getting pregnant after she and her boyfriend had unprotected sex, especially because she was still living at home. Her boyfriend bought her EC and she took the pills. The woman explained that she did not know about EC or what they would do, but her boyfriend had told her to take them because they would help her.

Following initial CDM use, the trajectories diverge and women's contraceptive experience differs based on their life circumstances.

#### Trajectory 1: Condom and EC

The first identified trajectory is a group of women who cycled between using condoms and EC in their contraceptive history (*n* = 7); for some women, they also relied on safe days or withdrawal to prevent pregnancy. The majority of these young women were still in school, all were nulliparous, and all were unmarried, but were mostly in stable dating relationships. All women in this trajectory used a condom as their first method and continue to use condoms intermittently. Most women used EC between one and three times, and went back to using condoms, albeit inconsistently in some cases, as their main contraceptive method.

Young women's influences on method choice for condoms and EC in this trajectory align with the reasons provided in the section on condoms and EC as a common starting point. The participants in this trajectory are exemplified by a general lack of knowledge about other contraceptive methods when they first used condoms or EC, yet continue to use these methods despite reporting knowledge of an average of about seven contraceptive methods at the time of interview. Many relayed that they first learned about condoms from teachers and friends, and two women said their boyfriends introduced them to condoms. After using condoms as their first contraceptive method type, women in this trajectory then used EC as their second method, mostly due to having unprotected sex or experiencing condom breakage. As with condoms, respondents in this trajectory mentioned several social influences on their choice to use EC, including friends, boyfriend and learning about EC at school. For both condoms and EC, women emphasized that the methods were easy to obtain, typically from the nearest shop or pharmacy, inexpensive and most often purchased by their boyfriends. Some respondents spoke about their increasing trust in the relationship, which led them to not use condoms every time they had sex:

*I was using a condom because I did not trust him as much since I had just met this person. But I stopped using it when I came to know more about him.”*

23-year-old, Nairobi, nulliparous

Two women in this trajectory expressed the desire to transition to a hormonal method of contraception. Despite desiring a long-acting method, one respondent cited fears about seeking a hormonal method due to concerns about unprofessional, unlicensed providers who may not provide full information or allow her to choose her method. The other respondent indicated that she would like to use an implant after a future first birth because the method had been effective for her mother at preventing pregnancy.

#### Trajectory 2: Condom to NCDM, for delaying first birth

Trajectory 2 is defined by women who begin using condoms and then transition to using a NCDM for reasons other than child spacing (*n* = 5). Only one woman reported periodic use of condoms after she transitioned to a NCDM. For the most part, these women were in committed relationships with a stable partner even prior to marriage with whom they openly discussed life plans, fertility goals and contraception. These respondents often refer to their desire to complete their education or their partner's education before becoming pregnant or having a child. These desires and goals led them to use contraception, and switch to methods that provided greater protection from pregnancy. Women in this trajectory initiated protected sex at an average age of 16, and at the time of interview remained nulliparous. Three were married. At the time of interview, two of the women in this trajectory had discontinued using contraception in order to get pregnant and one had done so due to infrequent sex.

Like women in the other trajectories, the respondents in this trajectory explained that they used condoms as their first contraceptive method for dual protection from HIV and pregnancy, and/or because of limited knowledge of other methods. However, women in Trajectory 2 also emphasized the importance of their education in their decision to use a condom. This sentiment is illustrated by this statement by a respondent who describes the decision to use a condom with her boyfriend as follows:

*I: Why did you decide to use the condom with your partner?*

*R: I decided to use it to prevent me from getting pregnant and diseases*.

*I: So when you met with your partner..*.

*R: I was still in school so if I got pregnant it would have disappointed my parents*.

21-year-old, Nairobi, nulliparous

Women in this trajectory switched to a NCDM at an average age of 17 and all considered themselves to be in a stable, long-term relationship. Most women first switched to injectables and one woman to the implant. Two of the injectable users then transitioned to implants. The respondents in the trajectory continued to prioritize education. In one example a nurse tried to discourage a young woman from using an implant and the young women said:

*I told her that am neither ready to get a child or to be married as at now since my partner is still in school. She asked me whether am faithful to my partner. I told her we have been in a relationship for so long and his parents are aware about it and my brothers know him too and that's why she decided to put it for me*.

22-year-old, Migori, nulliparous

Our analysis found that influences such as boyfriends or husbands and health care workers often influenced their decision to use the injectable or implant. Importantly, decision-making seemed to be undertaken jointly with their partners. A respondent explained the following about her decision to transition to the implant after experiencing side effects from injectables.

*I: And why did you choose to use the implant method?*

*R: According to the way the injectable… the way I saw the injectable is not good for my body, so I decided to try with the implant*.

*I: And who did you talk to when you were making that decision?*

*R: We made the decision the two of us. Me and my man*.

19-year-old, Mombasa, nulliparous

Decision-making about which NCDM to use was often guided by features of the method related to side effects, privacy or discreetness, duration of protection, and fears of infertility. Some women mentioned choosing the injectable instead of implants when they switched to a NCDM because the injectable lasted a shorter period of time and they wanted to become pregnant in the near future:

*I: Okay. And why did you decide to use Depo? Was there a specific reason why you chose Depo and not…*

*R: [Interjection] yeah. I didn't want to be injected for like the implant. Are they 5 or 3 years?*

*I: 3 or 5*

*R: [Interjection] so I didn't want that because I wanted a child too*.

*I: Oh. You wanted a short-term method because you wanted a child later*.

*R: Mmh*.

23-year-old, Nairobi, nulliparous

#### Trajectory 3: Condom to NCDM to EC

The third trajectory is characterized by unmarried women who switch between CDM and NCDM, primarily a result of being in short term relationships (*n* = 3). These respondents intermittently used condoms and EC throughout their contraceptive journeys, depending on the trust in their partners and whether or not they were currently using a NCDM. Because the relationships were, at times, short-term, women discontinued or changed the method they were using to correspond with infrequent sex. All of the respondents in this trajectory were unmarried at the time of interview and one of them had experienced a birth at the age of 15.

All three women in Trajectory 3 used condoms as their first contraceptive method, and then switched to a NCDM at some later point. The circumstances and factors that influenced respondents in the first part of their contraceptive journey were similar to the other trajectories that began with use of CDM. That is, they used condoms because it offered dual protection with partners they were in new relationships with, and whom they did not yet trust. Unlike some of the other trajectories, two respondents in this trajectory were influenced to use a condom by their friends and one decided on her own.

Two respondents subsequently switched to using implants and one to injectables. Both implant users changed from condoms to implants because they wanted a method of longer duration. One implant user and the injectable user also explained that they or their partners experienced side effects from the condoms. In one case, the respondent's partner was opposed to contraceptive use, so she used the implant discreetly and did not discuss with her sexual partner. All three women in this trajectory reported use of a condom concurrently with a NCDM at some point due to a lack of trust with their partner and one woman reported condom use when she was late for her re-injection. One respondent shared the following about her concurrent use of the implant and condoms:

*I: Why did you make that decision [to use condoms and implant concurrently]?*

*R: This one for three years… because I don't know the status of the person. You get me? I don't know if you are healthy or you are sick, that's when I decided…*

*I: [Interjection] and you did that how many times?*

*R: Many times. Because now if you meet someone and he doesn't want both of you to go to the hospital [to get an HIV test], you have to use Trust (a condom brand). Because, a child I have already prevented. I can't get pregnant, because I have prevented. What will you prevent AIDS with?*

24-year-old, Mombasa, 1 child

Two women went on to use EC after discontinuing a NCDM because of a change in relationship status followed by subsequent infrequent sex before initiating a new relationship. In the new relationship, the women did not immediately return to a healthcare provider to get another injection or implant, and instead used EC, often repeatedly, to prevent pregnancy after having unprotected sex. A 23-year-old woman from Migori explained that she used EC with her boyfriend because he lived far away and they had sex infrequently, and therefore did not need to use an injectable as she had previously. Additionally, two respondents experienced side effects with their NCDM and therefore desired a different, shorter-term method.

#### Trajectory 4: CDM to NCDM, after childbirth

Trajectory 4 is defined by women's transition from using a CDM to using NCDM methods after a pregnancy or birth (*n* = 9). Seven of these women used condoms and two used EC as their first method and the influences around first use are similar to those described under the section on a common starting place for contraceptive use. After using condoms and/or EC, the respondents eventually switched to using NCDM as their second or third method, primarily for birth spacing. Though all women transitioned to an NCDM after pregnancy/childbirth, the intentionality of the birth and the partner with whom the birth was with differed amongst women in this trajectory; about half of the respondents had a stable partner throughout their contraceptive history with whom they had a child (planned or unplanned) whereas the remaining respondents had an unplanned birth with a partner and went on to marry and have children with a different partner. About one-third of respondents used condoms concurrently with at least one of her NCDM because of a lack of trust in her relationship or wanting a back-up method. At the time of interview, all women in this trajectory had transitioned to marriage and had an average of 1.6 children (range 1–3).

For women in this trajectory, the primary impetus for switching from a CDM to a NCDM was for child spacing. Women often spoke of the need to allow their recently born baby to grow and develop before giving birth to another child. A respondent from Mombasa who had one child at the time of the interview shared:

*I have to take some time for my child to grow up to some stage; you know the child will have poor health in case I give birth again before she grows to some stage*.

22-year-old, Mombasa, 1 child

Another woman with two children at the time of the interview stated:

*Giving birth quickly is what I didn't want. I wanted my family to [be] spaced*.

23-year-old, Nairobi, 2 children

Women also shared secondary reasons for switching from CDM to NCDM, including wanting a longer-acting method and wanting to avoid another pregnancy to complete school.

Women were often influenced to use a NCDM method by a family member, such as a sister or mother, who shared their knowledge of and experience with a method with the women; or a health care provider who informed the women of FP methods during antenatal or postnatal care visits. Most women talked about either informing or discussing with their partners that they were going to start using a NCDM, but husbands were often secondary influencers with regard to these methods. In some cases, women used these methods covertly. One woman shared:

*I did not tell him. You know men disagree with these family planning issues. You know some women hide to go and get the methods of family planning, but once a man knows, you will see. They refuse, they dislike them. I don't know why. So I didn't tell my fiancé…I didn't tell him because had I told him he would have advised me to stop*.

21-year-old, Migori, 1 child (died at 2 months)

Respondents in this trajectory who switched from one NCDM to another often did so because of method related characteristics such as real or perceived side effects from, or myths about, the method; this was common in all trajectories where there was switching between NCDMs. This switching behavior was sometimes prompted by a health care provider. For example, after using injectables for 3 months for birth spacing after having her first child, a 21-year-old respondent from Mombasa recalled going back for her second injection and being told by the provider that she should “change from this injection to implant because the injection has side effects of not getting a baby completely,” indicating that the provider believed injections may lead to infertility.

The majority of respondents were using a NCDM at the time of interview. A small number of respondents stopped using contraception altogether by the time of the interview, largely due to the desire to conceive another child.

#### Trajectory 5: NCDM only, after childbirth

Trajectory 5 is comprised of six young women who only used NCDM in their reproductive history. The majority of the respondents were married (one was with a boyfriend who went on to become her husband) and all had one child when they first used contraception. Among the respondents in this trajectory, four started with the injectable and two started with oral contraception. Two respondents used a condom concurrently with their NCDM to protect against STIs due to a lack of trust in their relationship. Most of these women learned about the contraceptive method they chose as their first method from health care providers. Some learned about FP options during their antenatal care visits, or while bringing their infant to the clinic for check-ups.

Most of these women shared that they chose a NCDM first because they had an infant and wanted a method to space their next child. When asked about why she chose to use the injectable as her first contraceptive method, a respondent replied:

*So that I would not get pregnant before my child has completed breastfeeding*.

23-year-old, Mombasa, 2 children

Some women relayed their desire for child spacing to health-related reasons for both the infant and themselves, stating that it was good to rest from pregnancy and childbirth for some time, and better for the child to grow more before getting pregnant again. Relatedly, some women relayed their choice of first method to the duration of the method. Women who chose the injectable often shared that they wanted a “short-term method,” so that when they wanted to have their next child, they could stop using the method and have a return to fecundity without having to go to the clinic. One woman referred to the injectable as a “long-term method,” as she was comparing it to oral contraceptive pills and reported that she wanted a method that she did not have to remember to ingest every day.

Two women reported that their partners did not know that they were going to get contraception at the time of their first contraceptive method use. The remaining women shared that they informed their partners but there was no discussion, or they made the decision about a contraceptive method jointly with their husbands. One woman reported that she was heavily influenced by her partner regarding the type of contraception she first used.

All respondents switched to another NCDM as their second method, either from oral pills to injectables or injectables to implant, often related to the characteristics of the methods. Several women talked about forgetting to get their next dose of the method they were initially on, so chose another method that was longer acting. One woman who switched from using injectable as her first method to implants as her second method shared that she had been busy with work and forgot her injectable date and became pregnant. Another woman who also switched from injectables to the implant said that, after missing the date that she was supposed to get injected a few times, the service provider she went to recommended that she switch to the implant so she would not have to keep track of the date every 3 months, and she agreed. Women often followed the trajectory of oral pills to injectables to implants, which was heavily influenced by the counseling of health care providers.

Other women switched from one NCDM to another because of experiencing real or perceived side effects. One woman with three children adopted injectables, implants, and oral pills after the births of each of her children. She discontinued all three methods due to side effects from the methods, though ultimately decided that she would be willing to use oral pills in the future.

Some women often switched through a series of NCDM for a combination of reasons that included the fear of side effects and a desire for a longer-acting method. One young woman from Mombasa who had three children at the time of the interview decided to use oral pills as her first method when her first child was 3 months old. She initially wanted to use the injectable, but her husband preferred that she use oral pills, so she used the method for 6 months. After she started experiencing dizzy spells that she attributed to the method, she stopped using it and became pregnant soon after. After having her second child, she jointly decided with her husband that she would use the injectable. She used it for about a year, her husband recommended she stop using it because he believed the chemicals would cause infertility. She stopped using the injectable and became pregnant about 2 years later. After giving birth to her third child, she then had the 5-year implant inserted. The woman explained her decision to use the 5-year implant as follows:

*I wanted the child to grow. You know if you do not take care of yourself you can find out that you are pregnant again unexpectedly. That is why I decided to use the five-year method. You see I already have three children who are not well spaced, so I wanted to take a break and let these children grow up*.

24-year-old, Mombasa, 3 children

## Discussion

In this article, we identified five contraceptive use trajectories through the analysis of in-depth interviews with 30 young women from three counties in Kenya. Our study highlights how changing life circumstances and factors at multiple levels influence contraceptive decision-making during the adolescent and youth time periods. Additionally, we describe thematic areas that shape contraceptive choices over time. We also find that young women's relationship status and the occurrence of pregnancies or births are deeply intertwined with how various factors work to influence contraceptive method choice.

Our study found that condoms are an entry point to contraceptive use for many young women. The decision to begin their contraceptive journey with condoms was influenced by several factors, but overwhelmingly the most common influences were related to the strong desire for a method that protected against both pregnancy and STIs, easy accessibility and low cost at pharmacies and shops, and a lack of knowledge about other methods. Use of condoms may be driven, in part, by wider social acceptability of young people using condoms which is rooted in the desire for a method that is used only when needed (coital dependent), not perceived to impact future fertility, as well as the appreciation of the dual protection they provide (10, 15, 17). Our study found that young women learn about condoms from a variety of sources, including teachers, peers, their partner and parents; this differs from the paper from Ouma et al. which found that most young people learn about methods from peers and the internet (10).

Several of the trajectories highlight that young women who used two or more modern contraceptives in their life were able to successfully avoid a pregnancy. This is not limited to young women who are in stable relationships with one partner, but also includes women who may have short-term relationships. The contraceptive choices for trajectories 1–3, which are those young women who have avoided a pregnancy, include use of condoms and EC only, cycling through different types of CDM and NCDM, as well as women who made the choice to transition from a CDM to NCDM. This highlights that a range of contraceptive choices are available and appropriate for women to avoid pregnancy if desired and highlights the importance of ensuring young people are able to make an informed choice about which method they would like to use. The motivations for avoiding a pregnancy are somewhat different depending on the trajectory. Some women and (unmarried) couples value and emphasize their educational pursuits and therefore make informed, proactive decisions about contraceptive method choice, including the use of a hormonal method, in order to ensure that they avoid a pregnancy until they are ready.

For many young women, childbirth precipitated the transition to hormonal methods of contraception and this decision is often related to the desire to space births. Choice of method was often influenced by the health provider in the postnatal period and is typically decided through conversations between the woman, her partner (often husband) and the healthcare provider. As found in other studies in Kenya (15, 17), young women felt it was more acceptable to use hormonal methods after childbirth, which may be due in part to the pregnancy having alleviated personal and familial fears about future infertility. The majority of women did not transition back to condoms or EC once they had started using a hormonal method of contraception. The exception to this were women who did not trust their partner and used condoms with their hormonal method or women who used condoms as a stop gap measure between injections. Once using a hormonal method, many women tried more than one hormonal method which was driven by the desire to avoid/reduce side effects or find a method with characteristics that met their needs, such as having a long duration of protection.

This study is not the first to develop profiles of women and categorize their contraceptive needs and behaviors. Globally, the reproductive calendar from Demographic Health Surveys (DHS) has been utilized to explore how circumstances such as pregnancy, marriage or education influence contraceptive timing and method choice (22, 27, 38). In a DHS Analytical report (38), an analysis of data from Burundi categorized women of reproductive age into different segments based on patterns of contraceptive use, marriage and childbearing. The majority of young women fell into profiles of a “quiet calendar” with no contraceptive use and no pregnancies for the duration of the calendar or a “family builder” with no contraceptive use and two births for the duration of the calendar. Our study differs from these earlier studies in part because our sample is focused on experienced young contraceptive users and therefore does not include young women who align with the “quiet calendar” or “family builder” profiles. Further, our study focused on learning the experiences of young people and the stories surrounding their decision points. The thematic areas influencing contraceptive method choice in our study were similar to a recent paper by Igras et al. which used longitudinal qualitative data from Benin to explore the pathways women and men follow to meet their FP needs (25). Though similar high-level themes were influential on contraceptive behaviors, some themes, such as trust in the relationship, differed in our study due to the longitudinal perspective which captured influences on young women's reproductive choices prior to marriage or childbearing and our focus on contraceptive method choice rather than unmet need. While these earlier studies determined profiles of women and their behaviors, none provided significant depth on the dynamics of method choice among young women over their life transitions.

Each of the trajectories offers opportunities to develop different program strategies to support young women's access to and use of the contraceptive method of their choice while also aligning with the core principles underlying Kenya's 2015 National Adolescent Sexual and Reproductive Health (SRH) Policy (35). A common theme amongst study participants was that they had limited knowledge of contraceptive methods when they began their contraceptive journey. Relatedly, several young women said they learned about contraception from school or teachers. This points to and supports Kenya's 2015 policy to provide age-appropriate comprehensive sexuality education (CSE) to in-school and out-of-school youth. Yet, there are gaps in the content of CSE as shown by a three-county study in 2017 which found that <1 quarter of students were taught about different methods, how to use them or where to obtain contraception (39). In order for young women to be able to realize the benefits of contraception, it is critical that CSE includes information on a range of methods and provides further information on where young people can access health providers for individual-specific counseling. In addition to CSE, schools could link young women to SDPs that offer contraception to help young women access their method of choice. These components would help Kenya fulfill the 2015 SRH policy's guiding principle of being responsive to the varying needs of young women.

Several young women in this study mentioned the role and support of their parents as supportive of contraceptive use. The National Adolescent SRH Policy also highlights the critical role of parents in adolescent SRH and identifies the need to educate them on the SRH needs of young people, but does not elaborate on strategies to do so (35). Further work should explore how to reach parents of young people with information about contraceptive methods as well as provide strategies to parents on how to communicate with their children about contraception. Additionally, boyfriends and partners served as both supportive and prohibitive of contraceptive use and method choice. Those whom were supportive often were active decision-makers and helped to find a contraceptive method that suited their and their partner's needs. Given that young men are frequently active participants in the decision to use contraception, what method to use, and also are responsible for obtaining the methods, programs should focus on sensitizing men, young and old, about contraceptive methods and where to obtain them; this could include ensuring that young men receive information as part of CSE.

Among women who had at least one child, the provision of FP information at antenatal and postnatal care visits and the availability and access to postpartum FP methods are important strategies to help women choose and use a postpartum modern method (40). Our study found that receipt of FP information as part of the continuum of care often resulted in respondents transitioning to a longer-term method for birth spacing. Finally, given that many young women report obtaining their method from pharmacies, programs can also focus on expanding the range of methods available at pharmacies and ensure that young people know about this increased access. Kenya's 2019 policy allowing the provision of injectables at pharmacies, may be particularly helpful in expanding access and ensuring easier access to avoid lapses in method use (41).

### Limitations

This paper has several limitations. First, this study was designed to capture women's contraceptive journeys among a sample of young women who had used two or more types of modern methods. This allowed us to better understand the influences on multiple contraceptive decisions, but it also means that we are unable to extend these findings to women who have used fewer contraceptive methods. Additionally, because we did not interview young women who had not used contraception, we cannot fully discuss what makes the respondents in our study different from these women or tease out any factors that are barriers to contraceptive use.

Second, given the focus on contraceptive method choice, our interview guide did not ask details about the circumstances around first sex and subsequent sexual activity prior to women's first use of a contraceptive method. Therefore, there are gaps in understanding for some women and trajectories, such as the young women who did not start a contraceptive method until after they had given birth. It would be useful to have information prior to their first contraceptive use in order to fully unpack their reproductive journeys. Similarly, we did not ask detailed questions about number of sexual partners, so we are unable to systematically analyze the data by number of partners or type of partnership at each transition period.

Third, our inclusion criteria and interview guide also precluded any discussion of traditional methods. We did not specifically ask about use of traditional methods, so any mention of it came up organically in the interviews. Therefore, we are not able to fully understand the dynamics and transitions between traditional and modern method use during a young women's early reproductive history.

Fourth, our study may be subject to recall bias. We asked women ages 18–24 to recall their previous contraceptive use and decision-making. Women may not have been able to accurately recall events that happened several years in the past or the circumstances that influenced their decisions and behaviors.

Finally, this study was designed to explore influences on contraceptive method choice among young, female, experienced contraceptive users in Kenya. Given the qualitative design, these findings and the specific trajectories generated are not generalizable to the Kenyan population or to other countries and contexts. Undertaking this study in other settings would allow us to determine if similar trajectories are present and how they may vary in different settings.

## Conclusions

Our results indicate the diversity of contraceptive journeys or trajectories among a sample of young women who are experienced contraceptive users in Kenya. Despite many women beginning their contraceptive journey with condoms or EC, their experiences diverge and a variety of influences shape their subsequent contraceptive choices. For young women looking to delay their first birth, programs should explore expanding access to information and provision of NCDM prior to marriage through school-based programs, engagement of parents, and rolling out access in pharmacies.

## Data availability statement

Information about the study, survey tools and data are available at: https://dataverse.unc.edu/dataverse/fafc. A formal request needs to be made and a data sharing agreement will have to be made before sharing the data.

## Ethics statement

This study involving human participants was reviewed and approved by AMREF Health Africa Ethics and Scientific Review Committee (ESRC) (P205/2019), National Commission for Science, Technology and Innovation (NACOSTI) in Kenya, and the University of North Carolina at Chapel Hill Institutional Review Board (19-1360). Written informed consent for participation was not required for this study in accordance with the national legislation and the institutional requirements, but respondents voluntarily provided verbal informed consent to participate in the study. Additional approvals were secured from each county's Director of Health.

## Author contributions

This paper was conceptualized by LC, MM, BO, EW, CM, EZ, LB, and IS. LC, MM, BO, EW, and CM participated in study implementation and data collection. MM led data analysis with support from LC, BO, EW, and CM. TA, LB, TD, and IS provided input into the analysis approach and interpretation of data. LC and MM led the writing of this manuscript with critical input and revision done by BO, EW, CM, EZ, TA, LB, TD, and IS. All authors contributed to the article, approved the submitted version, and agree to be accountable for all aspects of the work.

## Funding

This work was supported, in whole or in part, by the Bill & Melinda Gates Foundation (INV-009814). Under the grant conditions of the Foundation, a Creative Commons Attribution 4.0 Generic License has already been assigned to the Author Accepted Manuscript version that might arise from this submission. We also received general support from the Population Research Infrastructure Program through an award to the Carolina Population Center (P2C HD050924) at the University of North Carolina at Chapel Hill.

## Conflict of interest

The authors declare that the research was conducted in the absence of any commercial or financial relationships that could be construed as a potential conflict of interest.

## Publisher's note

All claims expressed in this article are solely those of the authors and do not necessarily represent those of their affiliated organizations, or those of the publisher, the editors and the reviewers. Any product that may be evaluated in this article, or claim that may be made by its manufacturer, is not guaranteed or endorsed by the publisher.

## Author disclaimer

The contents of this article are solely the responsibility of the authors and do not necessarily represent the official views of the Carolina Population Center or the Bill & Melinda Gates Foundation.

## Abbreviations

AFIDEP: African Institute for Development Policy
BMGF: Bill & Melinda Gates Foundation
CDM: coitally dependent method
CSE: Comprehensive sexuality education
EC: emergency contraception
FAFC, Full Access: Full Choice project
FP: family planning
IDI: in-depth interview
I: interviewer
OC: oral contraception
IUD: intrauterine device
MOH: Ministry of Health
NCDM: non-coitally dependent method
NCPD: National Commission on Population and Development
NGO: non-governmental organization
R: respondent
SDP: service delivery point
SRH: sexual and reproductive health
UNC: University of North Carolina at Chapel Hill.
